# Factors associated with insomnia in older adult outpatients vary by gender: a cross-sectional study

**DOI:** 10.1186/s12877-021-02643-7

**Published:** 2021-12-07

**Authors:** Yu-Ting Peng, Ying-Hsin Hsu, Ming-Yueh Chou, Che-Sheng Chu, Chen-San Su, Chih-Kuang Liang, Yu-Chun Wang, Tsan Yang, Liang-Kung Chen, Yu-Te Lin

**Affiliations:** 1 Center for Geriatrics and Gerontology, Kaohsiung Veterans General Hospital, No. 386, Dazhong 1st Rd., Zuoying Dist., Kaohsiung City, 81362 Taiwan, Republic of China; 2 Department of Family Medicine, Kaohsiung Veterans General Hospital, Kaohsiung, Taiwan; 3 Division of Neurology, Department of Internal Medicine, Kaohsiung Veterans General Hospital, Kaohsiung, Taiwan; 4 Meiho University, Pingtung, Taiwan; 5 Aging and Health Research Center, National Yang Ming Chiao Tung University, Taipei, Taiwan; 6 Department of Geriatric Medicine, National Yang Ming Chiao Tung University School of Medicine, Taipei, Taiwan; 7 Department of Psychiatry, Kaohsiung Veterans General Hospital, Kaohsiung, Taiwan; 8 Department of Health Business Administration, Meiho University, Pingtung, Taiwan; 9 Center for Geriatrics and Gerontology, Taipei Veterans General Hospital, Taipei, Taiwan; 10 Taipei Municipal Gan-Dau Hospital, Taipei, Taiwan; 11 Department of Pharmacy, Tajen University, Pingtung, Taiwan

**Keywords:** Associated factors, Gender differences, Insomnia, Older adults

## Abstract

**Background:**

Insomnia is a common sleep disturbance in older adults and is associated with many poor health outcomes. This study aimed to explore factors associated with insomnia in older adult outpatient clinics, and to further analyze the influence of gender on factors associated with insomnia.

**Methods:**

This cross-sectional study was conducted in the outpatient clinics of a tertiary hospital in Southern Taiwan from July to September 2018. A total of 400 consecutive subjects aged 60 years or older were recruited. Insomnia was defined as a score of **≥**6 points on the Athens Insomnia Scale (AIS). Socio-demographics, health behaviors and clinical data were collected by face-to-face interview. Multivariable logistic regression was adopted for statistical analysis of the entire sample and stratified by gender.

**Results:**

Participants’ mean age was 74.74 ± 8.54 years, and the majority (93%) had more than one chronic disease. The prevalence of insomnia accounted for 30% (120/400) of all subjects, with males 22.9% (46/201) and females 37.2% (74/199). Gender, appetite, exercise, depressive symptoms, and sleep-related conditions such as short sleep duration, sleeping pills usage, medium-high risk of obstructive sleep apnea (OSA) and restless leg syndrome (RLS) were factors associated with insomnia in older adults. Exercise, sleeping pills usage, and RLS were independently associated with insomnia only in men, while appetite and medium-high risk of OSA were associated with insomnia in women only. In addition, after further adjusting for covariates, prevalence of the insomnia-related symptoms such as sleep induction, total sleep duration, sleep quality and sleepiness during the day was significantly higher in females than in males.

**Conclusions:**

Insomnia symptoms are highly prevalent among older adults, predominantly females. Significant differences are found between genders in factors associated with insomnia and insomnia-related symptoms. Understanding gender differences may help clinicians to modify associated factors when managing older adults with insomnia.

## Background

Since the world is facing an aging society, much attention has been paid to sleep problems in older adults [1]. Insomnia is a common sleep-related complaint among older adults, whether it is reported as subjective symptoms or defined as a sleep disorder according to recognized diagnostic systems. Although the definition varies by diagnostic criteria, about 30 to 48% of older adults are estimated to have insomnia symptoms, and the prevalence of insomnia disorder is around 12 to 20% [2]. Insomnia is not only associated with poor health outcomes such as cardiovascular disease [3], cognitive impairment [4], and mental disorders [5], but also reduces the quality of life [6] and poses a significant socioeconomic burden on society. Thus, finding potentially modifiable factors associated with insomnia is the first step in preventing insomnia-related consequences.

Evidence has revealed numerous factors associated with insomnia, including demographic, psychological and social characteristics. Female gender [7, 8], older adults who are divorced, separated or widowed [7], and those with lower levels of education and/or income, smoking, alcohol use, and reduced physical activity [7, 9] are factors associated with higher rates of insomnia in older adults. Clinical conditions such as physical illness [10], depression [11, 12], and some primary sleep disorders like obstructive sleep apnea (OSA) and restless leg syndrome (RLS) are also associated with or are comorbid with insomnia in various populations and clinical settings [2, 13, 14]. Nevertheless, the effects of insomnia on lifestyle and sleep behavior in older adults are not well established. Among the factors, female gender is frequently reported as a significant factor associated with insomnia [10, 11, 15–18]. However, information regarding gender differences in factors associated with insomnia is currently limited [19, 20]. Little is known about the influence of gender in factors associated with insomnia in older adults, too [18, 21].

To improve data collection for the symptoms of insomnia, we looked for factors that were potentially amenable to change among older adults. Data from a prior cross-sectional study that recruited participants from outpatient clinics of a tertiary hospital were used to investigate factors associated with insomnia in older adults. We further examined the differences in insomnia symptoms and factors associated with insomnia between genders.

## Methods

### Study population and data collection

This is a cross-sectional prospective study conducted at the Geriatric, Neurology, and Psychiatry outpatient clinics of Kaohsiung Veterans General Hospital (a tertiary medical center), Taiwan, from July to September 2018. Older adults with severe cognitive impairment (Clinical Dementia Rating 3 or above), psychiatric disorders (e.g., schizophrenia, bipolar disorder), or challenging communication were excluded. Most participants recruited from Psychiatry outpatient clinics had depressive symptoms, anxiety, insomnia, and dementia. For this study, we calculated the sample size based on the website (http://www.raosoft.com/samplesize.html), which used an error margin of 5%, confidence level of 95%, and an expected ratio of insomnia of 30% ~ 48% [2]. The range of results for expected sample size was 318 ~ 377. Physicians working in the outpatient clinics used convenience sampling to recruit participants on weekdays. Afterwards, face-to-face interviews were conducted by well-trained nursing researchers using structured questionnaires, including demographic data, personal lifestyle habits, physical and mood functions, and sleep-related characteristics. Finally, a total of 400 older adult subjects, aged 60-97 years, were recruited.

### Assessments

#### Demographic, lifestyle and clinical data

The study questionnaire for socio-demographic data included age, sex, educational level, marital and residential status, and occupational activities, collected through face-to-face interviews and chart-review. Simple dichotomous (yes-no) questions were used without further quantification for lifestyle factors, including current smoking status or alcohol drinking, coffee drinking, exercise habits, and afternoon naps. Appetite within the recent 3 months was investigated by asking each participant whether his/her appetite was good, fair or poor. Compared with poor appetite, we grouped participants with good or fair appetite into normal appetite. Among clinical data, obesity was defined as body mass index (BMI) ≥ 27 kg/m^2^ according to criteria listed by the Department of Health in Taiwan [22], since Asians seem to bear significant risks at lower BMI cut-offs than Caucasians [23, 24]. Data on chronic diseases and the diagnosis of dementia were reviewed from medical charts and drug lists, and self-reported by patients and their families. Chronic disease was defined as having any type of chronic disease for more than 3 months. We used the 5 items of the geriatric depression scale (GDS-5) to screen depressive symptoms [25, 26]. GDS-5 scores greater than or equal to 2 indicate abnormal results. Physical function was surveyed by baseline activities of daily living (ADL) based on the Katz Index (score range, 0–6: 0 = completely dependent to 6 = completely independent), and participants with scores below 6 were classified as dependent groups [27].

#### Definition of insomnia

Insomnia is defined by the Athens Insomnia Scale (AIS), a self-reported questionnaire designed to evaluate sleep difficulty. It consists of eight items, including sleeping time, sleep disruptions, premature awakening, total sleeping time, overall sleep quality, daytime physical and mental function, and daytime sleepiness. Each item is rated on a scale of 0-3 points (0 for no problems and 3 for severe insomnia-related symptoms). A cutoff value of **≥**6 points is used to define insomnia. The high reliability and accuracy of the AIS have been reported in previous validation studies [28], and the Chinese version validated in 2009 also showed high internal consistency [29]. In order to examine the relationship between each AIS item and gender, participants with any symptoms of each AIS item (score ≥ 1) were classified as having symptoms.

#### Sleep-related problems

Sleep-related problems, including sleep duration, sleeping pills usage, obstructive sleep apnea (OSA), and restless leg syndrome (RLS), were screened through yes/no questions or structured questionnaires. Self-reported actual duration of sleep (hours per night) within the last 1 month excluding the time in bed without sleep was recorded. The sleep duration was then grouped into long and normal sleep duration (> 6 h) and short sleep duration (≤ 6 h) [30]. STOP-Bang Sleep Apnea Questionnaire was used to screen for OSA. Scores of 0-2, 3-4, and 5-8 were categorized as low, moderate, and high risk for OSA [31, 32]. A four-question set recommended by the International Restless Legs Syndrome Study Group (IRLSSG) was used to define RLS [33]. Subjects who met all four criteria were classified into the RLS group.

### Statistical analysis

Socio-demographic and clinical data are presented as means +/− SD or frequencies and percentages. Comparisons were made between insomnia and non-insomnia groups using independent student t-test or one-way analysis of variance (ANOVA) for continuous variables and chi-square test for categorical variables. Factors that demonstrated a significant association (*p* < 0.05) with insomnia in univariable analysis were entered into multivariable logistic regression analysis to identify the independent relationship. Data were further stratified by gender, and analyzed using univariable and multivariable logistic regression analysis. The odds ratio (OR) and 95% confidence interval (95% CI) of each variable was documented. All data were analyzed using the SPSS version 22 (IBM, Armonk, NY, USA). Two-tailed *p*-values of < 0.05 were considered to be statistically significant.

### Ethical considerations

According to the principles of the Declaration of Helsinki, the study protocol was reviewed and approved by the internal review board of Kaohsiung Veterans Hospital, Taiwan (approval number VGHKS18-CT6-15). Participants were volunteers and all provided signed informed consent at the study inception. Participants also had the right to withdraw from participation at any time.

## Results

### Characteristics of study participants with or without insomnia

Among the 400 subjects who participated in the present study, the mean age was 74.74 years (SD, 8.54), and about one-half (49.8%) were women. A majority (93%) of participants had more than one chronic disease. Table 1 presents the differences in demographic information, life style, chronic diseases, physical function and sleep-related problems between participants with and without insomnia. The prevalence of insomnia defined by the AIS scale was 30% (*n* = 120), and was approximately 22.9% (46/201) and 37.2% (74/199) for male and female subjects, respectively. Subjects with insomnia had a significantly higher prevalence of poor appetite, not forming regular exercise habits, short sleep duration, tendency to use sleeping pills, depressive symptoms, and medium-high risk of OSA and RLS.

**Table 1:** Baseline characteristics of the study participants

	Total	Non-Insomnia (AIS < 6)	Insomnia (AIS ≥ 6)	
Characteristics	*n* = 400 (%)	*n* = 280 (%)	*n* = 120 (%)	*p* value
Age (60 – 97 years) ^a^	74.74 ± 8.54	74.88 ± 8.54	74.43 ± 8.55	0.630
Sleep duration (hrs)				< 0.001
≤ 6 h	221 (55.2)	131 (59.3)	90 (40.7)	
> 6 h	179 (44.8)	149 (83.2)	30 (16.8)	
Gender				0.002
Male	201 (50.2)	155 (77.1)	46 (22.9)	
Female	199 (49.8)	125 (62.8)	74 (37.2)	
Obesity (BMI > =27)				0.094
Yes	98 (24.5)	62 (63.3)	36 (36.7)	
No	302(75.5)	218 (72.2)	84 (27.8)	
Education				0.061
≤ 9 years (junior)	242 (60.5)	161 (66.5)	81 (33.5)	
≥ 10 years	158 (39.5)	119 (75.3)	39 (24.7)	
Marital status				0.161
Married	254 (63.5)	184 (72.4)	70 (27.6)	
Other	146 (36.5)	96 (65.8)	50 (34.2)	
Residential status				0.243
Live alone	42 (10.5)	28 (66.7)	14 (33.3)	
Live with others	351(87.8)	249 (70.9)	102 (29.1)	
Nursing home	7 (1.8)	3 (42.9)	4 (57.1)	
Occupational activity				0.355
Working	42 (10.5)	32 (76.2)	10 (23.8)	
Not working	358 (89.5)	248 (69.3)	110 (30.7)	
Current smoking				0.064
Yes	32 (8.0)	27 (84.4)	5 (15.6)	
No	368 (92.0)	253 (68.8)	115 (31.2)	
Current alcohol drinking				0.061
Yes	8 (2.0)	8 (100)	0 (0)	
No	392 (98.0)	272 (69.4)	120 (30.6)	
Drink Coffee				0.629
Yes	137 (34.2)	98 (71.5)	39 (28.5)	
No	263 (65.8)	182 (69.2)	81 (30.8)	
Appetite within 3 months				< 0.001
Fair	351 (87.8)	259 (73.8)	92 (26.2)	
Poor	49 (12.2)	21 (42.9)	28 (57.1)	
Exercise habits				0.015
Yes (Regular)	262 (65.5)	194 (74.0)	68 (26.0)	
No (non or irregular)	138 (34.5)	86 (62.3)	52 (37.7)	
Afternoon Nap				0.707
Yes	231 (57.8)	160 (69.3)	71 (30.7)	
No	169 (42.2)	120 (71.0)	49 (29.0)	
Sleeping pills usage				< 0.001
Yes	167 (41.2)	98 (58.7)	69 (41.3)	
No	233 (58.3)	182 (78.1)	51 (21.9)	
Any chronic disease^b^				0.060
Yes	372 (93.0)	256 (68.8)	116 (31.2)	
No	28 (7.0)	24 (85.7)	4 (14.3)	
Dementia (documented)				0.822
Yes	74 (18.5)	51 (68.9)	23 (31.1)	
No	326 (81.5)	229 (70.2)	97 (29.8)	
Activities of Daily Living				0.847
Total independence	365(91.3)	255 (69.9)	110 (30.1)	
Dependence	35 (8.2)	25 (71.4)	10 (28.6)	
Depression by GDS-5				< 0.001
No (< 2)	304 (76.0)	236 (77.6)	68 (22.4)	
Yes (≥ 2)	96 (24.0)	44 (45.8)	52 (54.2)	
Obstructive Sleep Apnea (risk)				0.004
Low	138 (34.5)	109 (79.0)	29 (21.0)	
Medium-High	262 (65.5)	171 (65.3)	91 (34.7)	
Restless Leg Syndrome				0.001
No	384 (96.0)	275 (71.6)	109 (28.4)	
Yes	16 (4.0)	5 (31.3)	11 (68.8)	

Abbreviations: *BMI* Body Mass Index, *GDS-5* Geriatric Depression Scale-5

^a^Continuous variables are presented as mean ± SD

^b^Chronic disease is defined as having any type of chronic disease for more than 3 months

#### Factors associated with insomnia in older adults

Table 2 shows the factors associated with insomnia determined by univariable and multivariable logistic regression analysis. In univariable logistic regression analysis, female gender, poor appetite within 3 months, without regular exercise, taking sleeping pills, medium-high risk of OSA, depressive symptoms, RLS and sleep duration ≤6 h have a significantly higher OR with insomnia. After adjusting all the covariates in Table 1 except sleep duration by multivariable logistic regression with forward stepwise method in Model 1, female gender (OR = 2.25, 95% CI = 1.33-3.82), poor appetite within 3 months (OR = 3.02, 95% CI = 1.53-5.95), using sleeping pills (OR = 1.96, 95% CI = 1.21-3.19), medium-high risk of OSA (OR = 2.99, 95% CI = 1.67-5.33), depressive symptoms (OR = 2.69, 95% CI = 1.58-4.56) and RLS (OR = 5.22, 95% CI = 1.66-16.40) were all independently associated with insomnia, except for non- or irregular exercise. When sleep duration was added into Model 2 as an additional covariate, the independent relationship between insomnia and those factors that were significant in Model 1 still existed, including female gender (OR = 2.35, 95% CI = 1.36-4.06), poor appetite within 3 months (OR = 3.87, 95% CI = 1.88-7.97), sleeping pills usage (OR = 1.90, 95% CI = 1.14-3.16), moderate-high risk of OSA (OR = 2.87, 95% CI = 1.56-5.26), depression (OR = 2.79, 95% CI = 1.59-4.87), and RLS (OR = 5.10, 95% CI = 1.52-17.14). In addition, short sleep duration (OR = 4.13, 95% CI = 2.38-7.17) was also an important independent factor associated with insomnia.

**Table 2:** Univariate and multivariate logistic regression of correlates of insomnia in older adults

			Model 1^b^		Model 2^c^	
Characteristic^a^	Unadjusted OR (95% CI)	*p* value	Adjusted OR (95% CI)	*p* value	Adjusted OR (95% CI)	*p* value
Gender (female)	2.00 (1.29-3.09)	0.002	2.25 (1.33-3.82)	0.003	2.35 (1.36-4.06)	0.002
Appetite within 3 months (poor)	3.75 (2.03-6.93)	< 0.001	3.02 (1.53-5.95)	0.001	3.87 (1.88-7.97)	< 0.001
Exercise (none / irregular)	1.73 (1.11-2.68)	0.015	–	–	–	–
Sleeping pills usage (yes)	2.51 (1.62-3.89)	< 0.001	1.96 (1.21-3.19)	0.006	1.90 (1.14-3.16)	0.013
OSA risk (Medium-High)	2.00 (1.24-3.24)	0.005	2.99 (1.67-5.33)	< 0.001	2.87 (1.56-5.26)	0.001
Depression (GDS ≥ 2)	4.10 (2.53-6.65)	< 0.001	2.69 (1.58-4.56)	< 0.001	2.79 (1.59-4.87)	< 0.001
RLS (yes)	5.55 (1.89-16.35)	0.002	5.22 (1.66-16.40)	0.005	5.10 (1.52-17.14)	0.008
Sleep duration (≤ 6 h)	3.41 (2.12-5.49)	< 0.001	–	–	4.13 (2.38-7.17)	< 0.001

Abbreviations: *OSA* obstructive sleep apnea, *GDS* Geriatric Depression Scale, *RLS* restless leg syndrome

^a^Only the significant variables in univariate logistic regression analysis are listed here

^b^Logistic regression analysis with the forward stepwise method in model 1 was adjusted for all factors in the Table 1 except for sleep duration

^c^Results in model 2 were derived from Model 1 with sleep duration added as another covariate

#### The influence of gender on factors associated with insomnia

After stratifying gender factors, univariate logistic regression analysis showed that in addition to female exercise, variables among all participants that were significantly associated with insomnia were also significantly associated with insomnia of male or female participants. After multivariable logistic regression analysis in model 1, only medium-high risk of OSA, depressive symptoms and RLS had the same significant association with insomnia in both genders (Table 3). Non- or irregular exercise (OR = 2.79, 95% CI = 1.31-5.92) and sleeping pills usage (OR = 2.65, 95% CI = 1.26-5.60) were independently associated with insomnia only in men, while poor appetite within 3 months (OR = 3.30, 95% CI = 1.42-7.70) was highly associated with insomnia in women only. In model 2, after controlling for sleep duration, the associations of medium-high risk of OSA (OR = 4.01, 95% CI = 0.97-16.66, *p* value = 0.056) in males and RLS (OR = 4.28, 95% CI = 0.99-18.59, *p* value = 0.052) in females lessened, and became only borderline significant.

**Table 3:** Gender differences in correlates of insomnia in older adults

	Male (*n* = 201)				Female (*n* = 199)			
	Model 1^b^		Model 2^c^		Model 1^b^		Model 2^c^	
Characteristic^a^	Adjusted OR (95% CI)	*p* value	Adjusted OR (95% CI)	*p* value	Adjusted OR (95% CI)	*p* value	Adjusted OR (95% CI)	*p* value
Appetite within 3 months (poor)	–	–	–	–	3.30 (1.42-7.70)	0.006	4.04 (1.66-9.85)	0.002
Exercise (none/irregular)	2.79 (1.31-5.92)	0.008	2.63 (1.20-5.78)	0.016	–	–	–	–
Sleeping pills usage (yes)	2.65 (1.26-5.60)	0.010	2.42 (1.11-5.31)	0.027	–	–	–	–
OSA risk (Medium-High)	3.91 (1.02-14.95)	0.046	4.01 (0.97-16.66)	0.056	2.90 (1.52-5.54)	0.001	2.64 (1.35-5.17)	0.005
Depression (GDS ≥ 2)	2.90 (1.25-6.72)	0.013	2.65 (1.08-6.54)	0.034	2.90 (1.48-5.69)	0.002	3.29 (1.62-6.68)	0.001
RLS (yes)	6.82 (1.00-46.34)	0.050	12.16(1.42-104.13)	0.023	5.27 (1.19-23.31)	0.028	4.28 (0.99-18.59)	0.052
Sleep duration (≤ 6 h)			4.91 (1.98-12.16)	0.001			3.56 (1.75-7.25)	< 0.001

Abbreviations: *OSA* obstructive sleep apnea, *GDS* Geriatric Depression Scale, *RLS* restless leg syndrome

^a^Only the significant variables in univariate logistic regression analysis are listed here

^b^Logistic regression analysis with the forward stepwise method in model 1 was adjusted for all factors in the Table 1 except for sleep duration

^c^Results in model 2 were derived from Model 1 with sleep duration added as another covariate

#### Associations between gender and insomnia symptoms

Table 4 discloses the associations between each AIS item and gender. Although the mean of each item of insomnia-related symptoms in female participants is higher than that in males, only “sleep induction,” “awakenings during the night,” “total sleep duration,” “sleep quality” and “well-being during the day” had statistically significant results. However, when we examined the OR of participants with insomnia-related characteristics, female participants had significantly higher ORs than males on the problems of sleep induction (OR = 2.32, 95% CI = 1.40-3.85), total sleep duration (OR = 3.57, 95% CI = 2.05-6.22), sleep quality (OR = 2.67, 95% CI = 1.57-4.54) and sleepiness during the day (OR = 1.72, 95% CI = 1.06-2.79).

**Table 4:** Associations between insomnia-related characteristics and gender

	Total	Male	Female		Total	Male	Female			
AIS items	*N* = 400	*N* = 201	*N* = 199	*p* value	*N* = 400	*N* = 201	*N* = 199	aOR^a^	95% CI	*p* value
AIS total scores	4.13 ± 4.39	3.19 ± 3.68	5.07 ± 4.84	< 0.001	120 (30.0%)	46 (22.9%)	74 (37.2%)	2.35	1.36-4.06	0.002
Sleep induction	0.74 ± 0.98	0.54 ± 0.87	0.93 ± 1.05	< 0.001	173 (43.3%)	68 (33.8%)	105 (52.8%)	2.32	1.40-3.85	0.001
Awakenings during the night	0.78 ± 0.96	0.68 ± 0.93	0.87 ± 0.98	0.049	188 (47.0%)	85 (42.3%)	103 (51.8%)	–	–	–
Final awakening	0.34 ± 0.68	0.29 ± 0.61	0.39 ± 0.75	0.130	95 (23.8%)	43 (21.4%)	52 (26.1%)	–	–	–
Total sleep duration	0.54 ± 0.91	0.34 ± 0.71	0.75 ± 1.03	< 0.001	128 (32.0%)	45 (22.4%)	83 (41.7%)	3.57	2.05-6.22	< 0.001
Sleep quality	0.61 ± 0.93	0.40 ± 0.72	0.82 ± 1.06	< 0.001	142 (35.5%)	54 (26.9%)	88 (44.2%)	2.67	1.57-4.54	< 0.001
Well-being during the day	0.41 ± 0.75	0.31 ± 0.66	0.51 ± 0.83	0.010	108 (27.0%)	43 (21.4%)	65 (32.7%)	–	–	–
Functioning capacity during the day	0.24 ± 0.58	0.20 ± 0.54	0.28 ± 0.62	0.183	69 (17.3%)	29 (14.4%)	40 (20.1%)	–	–	–
Sleepiness during the day	0.48 ± 0.79	0.44 ± 0.75	0.51 ± 0.83	0.343	128 (32.0%)	62 (30.8%)	66 (33.2%)	1.72	1.06-2.79	0.029

^a^Reference group: male; Logistic regression analysis with the forward stepwise method was adjusted for all factors in the Table 1

## Discussion

The present cross-sectional study targeted a total of 400 older adult participants in outpatient clinics to comprehensively investigate the factors associated with insomnia, especially considering the influence of common sleep-related problems such as sleep duration, sleep medication usage, OSA and RLS, and intending to find the influence of gender on the associated factors. Results of the present study showed that gender, poor appetite, not exercising regularly, depressive symptoms, and sleep-related conditions such as short sleep duration, sleeping pills usage, medium-high risk of OSA and RLS were factors associated with insomnia. Gender was also found to influence partially associated factors for insomnia. Exercise, sleeping pills usage, and RLS were relevant to insomnia in males alone, whereas poor appetite and medium-high risk of OSA were relevant to insomnia in females only. Otherwise, significant associations were found between short sleep duration, and depressive symptoms and insomnia in both genders.

The prevalence of insomnia depends on the method of identification. In the present study, the prevalence of AIS-defined insomnia was 30% for all subjects and is similar to the reports in previous studies, which ranged from 30 to 48% [2]. In addition, we found that around three-quarters of participants had at least one symptom of AIS (301/400, 75.2%; male 141/201, 70.1%; female 160/199, 80.4%). Consistent with previous studies [10, 11, 15–18], females have a higher percentage of insomnia than males, and we also reported significantly higher scores in females than in males even after controlling for multiple potential confounders. In a meta-analysis, females had a 41% increased risk for having insomnia than males [34]. In addition, for older adults aged 65 and over, the risk increased to 73%. Hormonal changes such as estrogen and progesterone levels after menopause play an important role in the sleep of older females [35]. Females are also likely to suffer from pain-related physical conditions such as osteoporosis, back problems, and fracture, and psychiatric problems such as depression and anxiety [36, 37]. Higher risk of insomnia among females may be associated with these conditions. The definition of insomnia used in previous studies is usually derived from the three key symptoms of insomnia, including difficulty initiating sleep, difficulty maintaining sleep, and early morning awakening. The prevalence of these symptoms is estimated to be 4.5-16.4%, 6.5-23.1% and 4.0-16.7%, respectively [19–21, 38, 39]. In the present study, the prevalence of sleep induction (43.3%), awakening during the night (47.0%) and final awakening (23.8%) in AIS, which is similar to the three key symptoms of insomnia, were obviously higher. However, different scoring methods may affect prevalence rates. The AIS, which was used to define insomnia in the present study, recorded the severity of insomnia symptoms rather than the frequency of insomnia symptoms used in previous studies. In addition, regarding isolated insomnia-related symptoms, current reported results have been inconsistent [20, 21]. La. et al. [20] reported no obvious gender differences of difficulty initiating sleep, difficulty maintaining sleep, and early morning awakening for community-based Koreans aged 19 to 69 years. However, Jaussent et al. [21] recruited 5886 community-dwelling adults aged 65 or older in three French cities, and the results showed that, although difficulty maintaining sleep was the most common symptom, the proportion of women who had difficulty initiating sleep was significantly higher than that of men. In the present study, after further adjusting for covariates, sleep induction (similar to difficulty initiating sleep) was significantly more prevalent among females than in males rather than awakenings during the night and final awakening. In addition, the insomnia-related symptoms such as total sleep duration, sleep quality and sleepiness during the day have also been found to be significantly associated with females, and should be further explored in future study.

In the present study, there was no tendency to have higher prevalence with aging, even though after adjusting for covariates (age 60-65y/o 32.8%; 66-70y/o 29.4%; 71-75y/o 26.8%; 76-80y/o 30.1%; 81-85 y/o 35.7%; 86-97 y/o 25.5%). Age is considered to be a common risk factor of insomnia; however, the results are not consistent when the studies focused on older adults. Wang et al. [18] recruited 2195 community-dwelling participants in a cross-sectional survey in rural China, finding that age was not significantly correlated with insomnia after controlling for sex, marital status, education, occupation and chronic diseases. Another community-based study in China found that, compared with participants younger than age 65, only participants aged 75 and over had a significantly higher risk of insomnia [38]. Current smoking, alcohol drinking and chronic diseases were also not found to be associated with insomnia in the present study. Recently, a meta-analysis and a large-scale community-based cohort study demonstrated that smoking and higher alcohol consumption was associated with poorer sleep quality and insomnia [40, 41]. However, neither of these two studies investigated the subgroup of older adults, even though the population in both studies included older adults. In contrast, a study of participants aged 60 and over showed that men who drank occasionally had lower insomnia risk than men who drank frequently [38]. The prevalence of smoking and alcohol drinking among older adults varies between study populations. In our study, we only identified 8% of current smoking and 2% of current drinking, and the small sample size may be a possible explanation for the absence of statistical significance. Relationships between insomnia and some chronic diseases such as coronary artery disease, chronic obstructive pulmonary disease, or cerebral hemorrhage have been reported [18, 38]. However, regardless of the insomnia or non-insomnia group, participants recruited from outpatient clinics in the present study, had a higher percentage of having one and more chronic diseases, and having a high percentage of chronic diseases in both groups may lessen the influence of chronic diseases on insomnia. Although some epidemiologic studies reported a negative association between regular exercise and insomnia, after adjusting only for demographic data and mood symptoms [42, 43], the present study also did not find similar significant associations after evaluating the effects of sleep-related problems. In addition, based on results of studies using quantitative exercise measurement instead of subjective self-reported questions as in the present study, both physical activity of moderate intensity and exercise training programs obviously improved sleep symptoms and quality [44, 45].

Sleep-related problems such as OSA, RLS, short sleep duration and sleeping pills usage, are highly correlated with insomnia, but are rarely reviewed simultaneously in one study. After considering all the influencing covariates, we found that short sleep duration, sleeping pills usage, medium-high risk of OSA and RLS were all independently associated with insomnia. Although the information of sleep duration was collected by questionnaires, the significant results were similar to the results derived from the general population using objective measures such as polysomnographic studies [46]. OSA and RLS both increase the prevalence and severity with advancing age [47], and are frequently comorbid with insomnia. OSA is characterized by repeated partial or complete obstruction of the respiratory tract and impaired ventilatory control during sleep, and therefore, were responsible for consequences such as interrupted sleep, insomnia, daytime sleepiness, neurocognitive impairment and cardiovascular diseases [13, 48, 49]. Older adults tend to take sleeping pills to relieve sleep complaints, however, the present study disclosed that those who use sleeping pills still tend to complain of insomnia symptoms. Few studies have focused on older adults who use hypnotic agents, except for those with insomnia. Although hypnotic agents improve sleep duration and latency, the magnitude of effect from sleep medications is small and potentially increases various adverse effects [50, 51]. In addition, a variety of sleep medications have different mechanisms of action and different clinical effects on insomnia, which may influence patients’ subjective feelings regarding sleep. Previous studies of young to youngest older adult participants have shown that demographic information, occupational and lifestyle factors are associated with the risk of insomnia, while we found that sleep-related problems in older adults are more associated with insomnia. The prevalence of sleep-related problems in older adults increased significantly. Therefore, compared with other factors, sleep-related problems may be a factor that needs more attention among older adults with insomnia.

In particular, the present study is the first to identify the significant relationship between poor appetite and insomnia. Some clinicians have attributed poor appetite to be a manifestation of depression, but in the present study, the effect still existed in multivariable logistic regression analysis after adjusting for depressive symptoms identified by GDS-5. In addition, poor appetite may result in poor nutritional status, and previous studies have shown that nutritional inadequacy and malnutrition status identified by the Mini Nutritional Assessment (MNA) scores correlate highly with insomnia [52, 53]. Furthermore, poor appetite may reduce the consumption of foods rich in melatonin, a hormone secreted by the pituitary gland that is part of the sleep-wake cycle and is reported to improve sleep efficiency [54]. In addition, the effects of depression on insomnia remained strong across genders in the present study after controlling for covariates, including sleep-related problems. A review of prospective studies has also recognized depression as a risk factor for sleep disturbances [11]. Various mechanisms have been proposed to help explain the findings relative to the effects of depression on sleep, including inflammatory regulations, genetic and familial factors, and the effects of social and environmental factors [55].

Although gender differences in insomnia represent a complex interaction between biological, psychological and social factors [56], another key question that needs to be answered is whether the high prevalence of insomnia in older females is affected by different factors associated with insomnia in males and females. Gender not only influences the prevalence of insomnia, but a few studies suggest that it also may significantly affect the factors associated with insomnia in older adults. However, most studies are mainly for community-dwelling participants and not for older adults. A study of insomnia, defined by any single symptom of difficulty initiating sleep, difficulty maintaining sleep, and early morning awakening, in 9851 Chinese adults aged 18-65 in Hong Kong revealed that socio-demographic factors, medication usage, and minor psychiatric disturbance (e.g., depression and anxiety) associated with insomnia were significantly different between genders [19]. Lower education level and being retired are associated with insomnia in males, and being a housewife, divorced or widowed and complaining of a nocturnal noisy environment were associated with a higher risk of insomnia in females. Another study of 2695 participants aged between 19 to 69 years old in the Korean general population, however, found that both genders shared the same risk factors of short sleep duration, anxiety and depression with insomnia (the Insomnia Severity Index), but for males, low income was significantly associated with insomnia [20]. The present study is in line with that study’s report that the associations between insomnia and most demographic data and lifestyle factors were lessened by depression and sleep-related problems. Two community-based studies in China and France investigated factors associated with insomnia in older males and females. The French study showed that hormone replacement therapy, Mediterranean diet, caffeine and alcohol intake had a protective effect on older women, while another Chinese study showed that there are specific insomnia susceptibility factors between older men and women, especially targeting individual chronic diseases (coronary heart disease for males; cerebral hemorrhage, hyperlipidemia, and mild cognitive impairment for females) [21, 38]. However, neither of those two studies considered sleep-related factors in the analysis, such as sleep duration, OSA or use of sleeping pills. In our study, insomnia was defined by AIS, which is different from previous studies. After controlling for all study variables, except for sleeping pills usage in females, both genders shared the same risk factors for sleep-related problems as all participants. In further analysis, we found that the association between sleeping pills usage and insomnia disappeared after adding depressive symptoms into the model. Higher rates of using sleeping pills in older females with depression compared to older males (65.6% vs. 57.1%), was also noted. Higher rates of using sleeping pills in depressive females may explain why depression alters the association between sleeping medication and insomnia. The findings in another study, which found that sleeping pills usage was only related to difficulty in initiating sleep, may explain why there was no significant association [20]. Besides sleep-related problems, gender-specific risk factors still exist. We found that exercise was shown to be associated with insomnia in males alone, whereas appetite was associated with insomnia in females only. Kline. et al. [57] conducted a clinical review of consistent effects of physical activity on sleep outcome across age and gender. However, the evidence was mixed among older adults. In addition, another systematic review and meta-analysis of randomized controlled trials for evaluating the effect of exercise on sleep quality and insomnia in middle-aged women, reported that moderate levels of programmed exercise rather than low levels of physical activity had a positive effect on sleep quality, but physical activity didn’t show an effect on reducing the severity of insomnia [58]. Older females had higher risk of suffering from depressive symptoms, and changes in appetite was an important symptom for which patients with depression searched for help [59]. Therefore, poor appetite may help to account for a higher risk of insomnia in females. According to our results, when managing insomnia for men, we should consider assessing their physical activity and recommend regular exercise, while for women, we need to pay attention to nutritional status and screen for appetite.

## Study contributions, implications, limitations

The present study evaluated a group of older adult outpatients with a high prevalence of insomnia, and identified factors associated with insomnia, summarizing some potentially modifiable factors for further intervention. Evaluation with validated questionnaires provided conceivable results that could be followed and compared with other populations. Substantial differences found between genders in factors associated with insomnia added new perspectives in clinical practice.

Nevertheless, the study has several limitations. First, the cross-sectional design limits drawing causative conclusions, and sleep patterns were not evaluated by objective measures such as polysomnography or actigraphy. Second, collection of socio-demographic variables based on self-reported data or simple dichotomous yes/no questions may result in recall bias, especially among this older adult population, and there may still be some potential confounding factors left behind. Since the diagnosis of RLS was based on the four screening criteria of the IRLSSG, some mimicking diagnoses may be inevitably included. Third, subjects were recruited from outpatients in a single medical center and thus results may not be extrapolated to different settings or areas.

## Conclusions and future study

The prevalence of older adults with insomnia symptoms is high, affecting about one in three older outpatients. Female gender, appetite, depression, and some sleep-related conditions (short sleep duration, OSA, RLS, and sleeping pills usage) are significantly associated with insomnia. Significant differences are found between genders in factors associated with insomnia. Understanding gender differences identified in this study may help clinicians to modify associated factors when managing older adults with insomnia.

## Data Availability

The datasets analysed during the current study are available from the corresponding author Chih-Kuang Liang on reasonable request. Please contact (ck.vghks@gmail.com) for data access.

## Abbreviations

AIS: Athens Insomnia Scale
BMI: Body Mass Index
GDS-5: Geriatric Depression Scale-5
IRLSSG: International Restless Legs Syndrome Study Group
MNA: Mini Nutritional Assessment
OSA: Obstructive sleep apnea
RLS: Restless leg syndrome
